# Enzymatic process optimization for the in vitro production of isoprene from mevalonate

**DOI:** 10.1186/s12934-016-0622-4

**Published:** 2017-01-09

**Authors:** Tao Cheng, Hui Liu, Huibin Zou, Ningning Chen, Mengxun Shi, Congxia Xie, Guang Zhao, Mo Xian

**Affiliations:** 1 CAS Key Laboratory of Bio-based Materials, Qingdao Institute of Bioenergy and Bioprocess Technology, Chinese Academy of Sciences, No. 189 Songling Road, Laoshan District, Qingdao, 266101 China; 2 College of Chemical Engineering, Qingdao University of Science and Technology, Qingdao, 266042 China; 3 State Key Laboratory Base of Eco-Chemical Engineering, College of Chemistry and Molecular Engineering, Qingdao University of Science and Technology, Qingdao, 266042 China

**Keywords:** Mevalonate, Isoprene, Synthetic biochemistry, Isoprenoids, Bio-based chemicals, Enzymatic process

## Abstract

**Background:**

As an important bulk chemical for synthetic rubber, isoprene can be biosynthesized by robust microbes. But rational engineering and optimization are often demanded to make the in vivo process feasible due to the complexities of cellular metabolism. Alternative synthetic biochemistry strategies are in fast development to produce isoprene or isoprenoids in vitro.

**Results:**

This study set up an in vitro enzyme synthetic chemistry process using 5 enzymes in the lower mevalonate pathway to produce isoprene from mevalonate. We found the level and ratio of individual enzymes would significantly affect the efficiency of the whole system. The optimized process using 10 balanced enzyme unites (5.0 µM of MVK, PMK, MVD; 10.0 µM of IDI, 80.0 µM of ISPS) could produce 6323.5 µmol/L/h (430 mg/L/h) isoprene in a 2 ml in vitro system. In a scale up process (50 ml) only using 1 balanced enzyme unit (0.5 µM of MVK, PMK, MVD; 1.0 µM of IDI, 8.0 µM of ISPS), the system could produce 302 mg/L isoprene in 40 h, which showed higher production rate and longer reaction phase with comparison of the in vivo control.

**Conclusions:**

By optimizing the enzyme levels of lower MVA pathway, synthetic biochemistry methods could be set up for the enzymatic production of isoprene or isoprenoids from mevalonate.

## Background

Commodity chemicals are traditionally produced from petroleum via the energy-intensive chemical methods. Alternative process that does not rely on the petroleum resources and chemo-process is under fast development for chemical production, using the advanced tools of synthetic biology and metabolic engineering [1, 2]. Recently, isoprene and a variety of isoprenoids can be produced from renewable feedstock through engineered microbes [3–7]. The general strategy is engineering the whole isoprenoid biosynthesis pathways in the chassis strains [6], and the mevalonate (MVA) pathway other than the methylerythritol 4-phosphate (MEP) pathway is usually selected to construct robust strains with higher production titers [8].

However, the biosynthesis of isoprene and isoprenoids by the cell-based strategy faced one major bottleneck: the nutrient limitation and the accumulation of toxic intermediates or products may lead to cell growth inhibition and limited yields and titers; thus rational engineering and optimization are often demanded to make the process economically feasible [9–12]. Corresponding synthetic biochemistry tool has also been demonstrated to overcome the major bottleneck of the cell-based strategy, especially in rational optimization of synthetic multi-enzyme pathways [13]. The emergence of cell-free system has several advantages over the cell-base system: (1) process can be operated continuously; (2) can synthesize hazard products which are toxic towards living cell system; (3) enzyme can be quantitatively and biochemically adjusted to optimize flux through the metabolic pathways; (4) reduce the operation (like gas-stripping) and purification requirements; (5) higher yields without competing cellular pathways. In case of isoprene biosynthesis, a recent cell-free approach for the conversion of the glycolysis intermediate phosphoenolpyruvate into isoprene at nearly 100% molar yield was promoted [14]. The in vitro system involves enzymes through the full MVA pathway. While challenges also exist for the synthetic biochemistry platform, as the cofactors of ATP, NADPH, and acetyl-CoA need to be balanced in the complex MVA pathway: NADPH and acetyl-CoA are involved in the upper MVA pathway (from pyruvate to MVA), ATP is involved in the lower MVA pathway (from MVA to isoprene). Thus additional enzymes are needed to balance the supply/consumption of these co-factors which increased the total enzymes of 12 in the complex in vitro system for bioisoprene [13, 14].

This study focused on implementing an enzymatic process only consisting of lower MVA pathway for the biosynthesis of isoprene directly from MVA. In the demonstrated system, the cofactors of NADPH and acetyl-CoA are not involved, which decreases the needs of additional enzymes to balance these factors. Moreover, the enzyme activities and quantities of the simplified system (only involves 5 enzymes) can be easily and precisely adjusted to optimize flux through the biochemical steps, which would improve the conversion efficiency comparing to the current in vitro platform for bioisoprene.

## Results and discussion

### Purification of the enzymes in lower MVA pathway

**Fig. 1:**
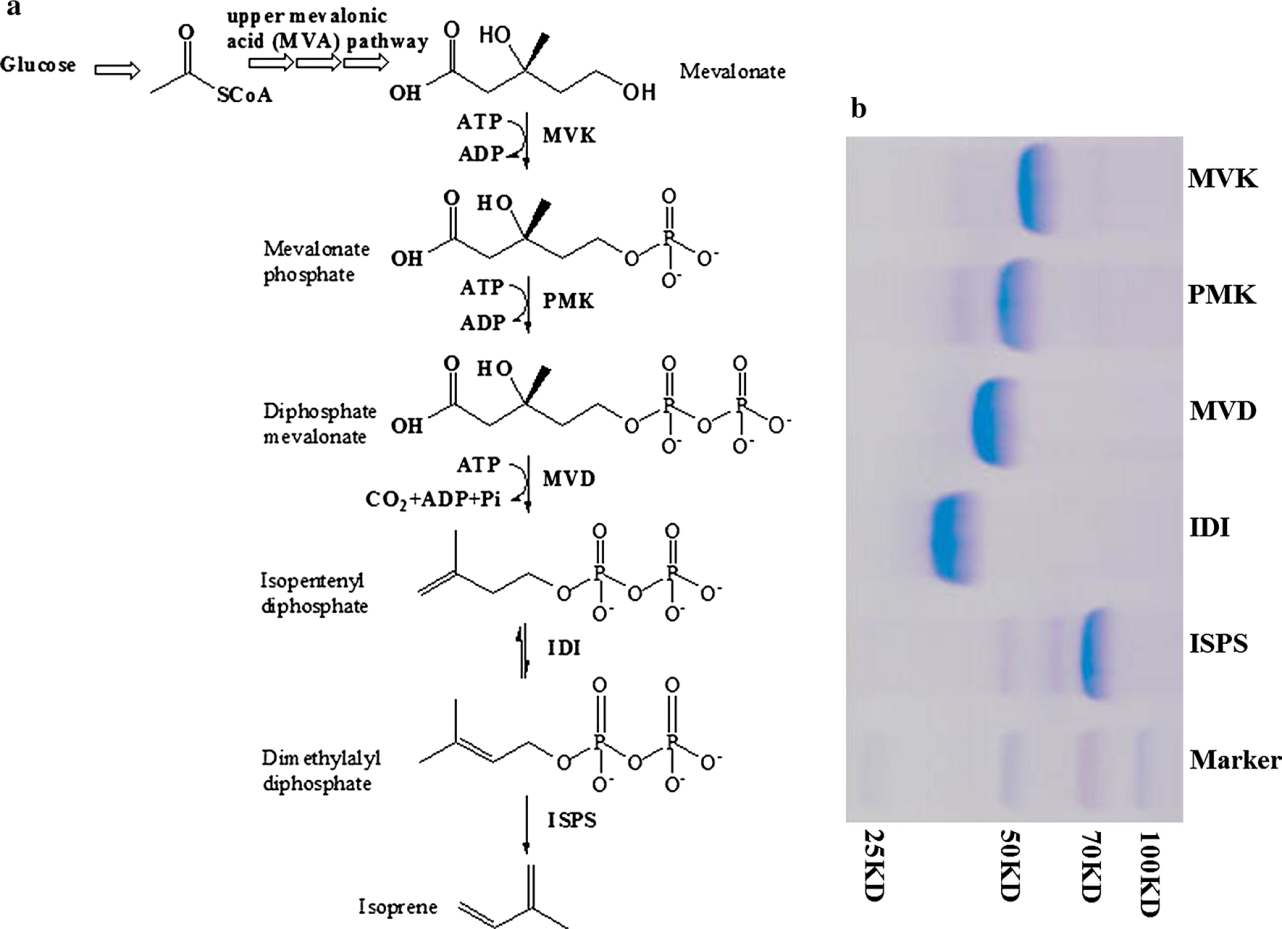
The purified enzymes of the lower MVA pathway. **a** Pathway overview. The lower MVA pathway consists of 5 enzymatic reactions each catalyzed by MVK (mevalonate kinase, EC 2.7.1.36), PMK (phoshpomevalonate kinase, EC 2.7.4.2), MVD (diphoshpomevalonate kinase, EC 4.1.1.33), IDI (isopentenyl diphosphate isomerase, EC 5.3.3.2) and ISPS (isoprene synthase, EC 4.2.3.27). **b** The size of the purified enzymes

In this study, four biosynthetic enzymes of lower MVA pathway and isoprene synthase were individually prepared by recombinant *E. coli* strains (Table 1) and purified before in vitro production. Through these enzymes mevalonate can be converted to dimethylallyl pyrophosphate (DMAPP) by primary mevalonate kinase (MVK, EC 2.7.1.36) and secondary phosphorylation (phosphomevalonate kinase, PMK, EC 2.7.4.2), decarboxylation (diphosphomevalonaet decarboxylase, MVD, EC 4.1.1.33) and isomerization (isopentenyl diphosphate isomerase, IDI, EC 5.3.3.2), then the isoprene synthase (ISPS, EC 4.2.3.27) catalyzes the formation of isoprene from DMAPP. For each molecular of isoprene from MVA, 3 ATP is needed and NADPH is not demanded for the in vitro bioconversion, which is different with the in vivo system from pyruvate [14, 15]. In order to prevent the formation of inclusion body and ensure correct enzyme folding, each enzyme was purified and checked their molecular size by SDS-page before enzymatic assay experiments. We found that keeping the cultures at 20 °C after IPTG induction in the fermentation process helped to improve the yield of soluble recombinant enzymes. The purity and molecular size of the purified enzymes can be seen in Fig. 1.

**Table 1:** Bacterial strains and plasmids used in this study

Strain/plasmid/primer	Relevant genotype/property/sequence	Source/reference
Strains		
*E. coli* BL21(DE3)	F^−^ *ompThsdS*_*B*_(*r*_*B*_^−^ *m*_*B*_^−^) *gal dcm rne131* (*DE3*)	Invitrogen
MVK producer	BL21(DE3)/pET-ERG12	This study
MVD producer	BL21(DE3)/pET-ERG8	This study
PMK producer	BL21(DE3)/pET-ERG19	This study
IDI producer	BL21(DE3)/pET-IDI	This study
ISPS producer	BL21(DE3)/pET-ISPS	This study
Plasmids		
pYJM14	pTrcHis2B derivative carryinggenes *ERG8*, *ERG12*, *ERG19* and *IDI*, Trc promoter, Ap^R^	[16]
pET-ERG12	pET30a(+) derivative carryinggenes gene *ERG12*, T7 promoter, Kan^R^	This study
pET-ERG8	pET30a(+) derivative carryinggenes gene *ERG8*, T7 promoter, Kan^R^	This study
pET-ERG19	pET30a(+) derivative carryinggenes gene*ERG19,* T7 promoter, Kan^R^	This study
pET-IDI	pET30a(+) derivative carryinggenes gene *IDI,* T7 promoter, Kan^R^	This study
pET-ISPS	pET30a(+) derivative carryinggenes gene *ISPS,* T7 promoter, Kan^R^	This study
pACY-ISPS	pACYDuet-1 derivative carryinggenes gene ISPS, T7 promoter, Cm^R^	This study
Primers		
ERG12_F	5′-CCCAAGCTTGGTCATTACCGTTCTTAACTTC-3′	
ERG12_R	5′-CCGCTCGAGTTATGAAGTCCATGGTAAAT-3′	
ERG8_F	5′-CCGGAATTCTCAGAGTTGAGAGCCTTCAG-3′	
ERG8_R	5′-CCGCTCGAGTTATTTATCAAGATAAGTTT-3′	
ERG19_F	5′-CGCGGATCCACCGTTTACACAGCATCCGT-3′	
ERG19_R	5′-CCGCTCGAGTTATTCCTTTGGTAGACCAG-3′	
IDI_F	5′-CGCGGATCCACTGCCGACAACAATAGTAT-3′	
IDI_R	5′- CCGCTCGAGTTATAGCATTCTATGAATTT-3′

### In vitro production of isoprene from mevalonate

**Fig. 2:**
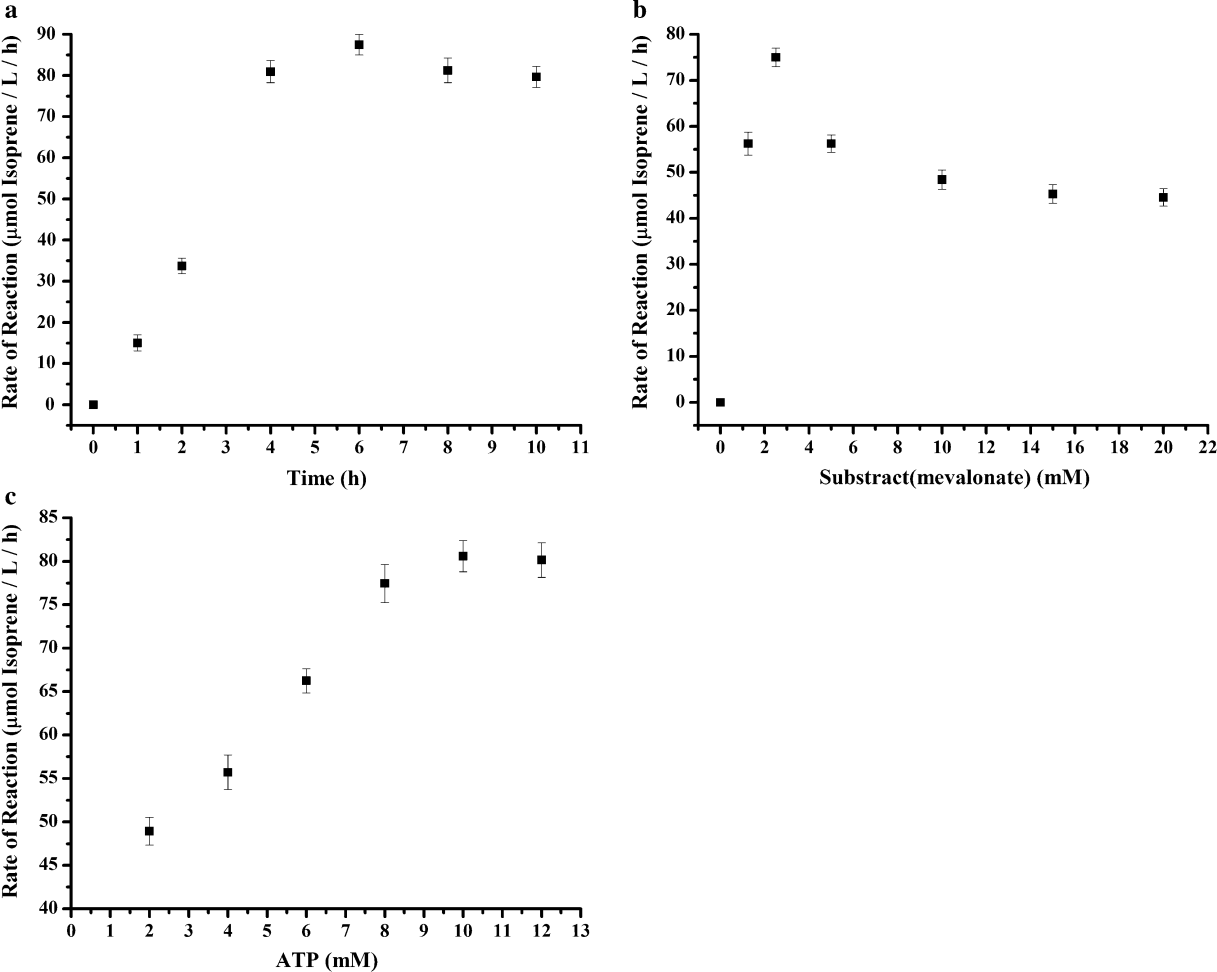
Determination of the optimal enzymatic conditions. The production rate of isoprene in 2 ml enzymatic system under conditions: **a** 2.5 mM mevalonic acid, 12 mM ATP, with varied reaction time; **b** 12 mM ATP, reaction for 4 h, with varied concentration of mevalonic acid; **c** 2.5 mM mevalonic acid, reaction for 4 h, with varied concentration of ATP. The data shown are means of three repeated experiments and the *error bars* present the standard deviation

With the purified enzymes, we further tested whether the lower mevalonate pathway can be reconstituted in vitro by mixing of substrate and ATP with the purified enzymes. The addition of ATP is compulsory as three ATP are used in the phosphorylation and decarboxylation of mevalonate to IPP (Fig. 1). In the 2 ml in vitro system with purified enzymes (0.5 µM each), substrate (2.5 mM) and ATP (12 mM), we took samples and analyzed total isoprene at different time points and calculate the rate of reaction (total isoprene/L/h) at the different time points. The results showed that the maximum isoprene production (76.5 µmol/L/h, 5.2 mg/L/h) occurred at 4 h after the addition of ATP and mevalonate (Fig. 2a), similar with the time course curve of in vitro isoprene production from PEP in the previous study [14].

We next tested the optimum mevalonate/ATP ratio in the 2 ml in vitro system. The optimum concentration of mevalonate (substrate) was analyzed by starting with a fixed amount of ATP (12 mM) and sequentially increasing amount of mevalonate (Fig. 2b). The results showed that the maximum isoprene production was achieved when 2.5 mM mevalonate was added in the system, and the isoprene production was decreased when the initial concentration of mevalonate was over 2.5 mM. We also tested the optimum concentration of ATP with fixed amount of mevalonate (2.5 mM). The results showed that supplementation of 10 mM ATP resulted in the maximum isoprene production (Fig. 2c). The production of isoprene was increasing with the increasing of ATP from 2 to 12 mM, it is suggested that the lower pathway of MVA was inhibited when the concentration of substrate of mevalonate was higher than 2.5 mM. The optimum ratio for substrate (mevalonate) and cofactors (ATP) is around 1:4, which is excess to the theoretical ATP consumption (1:3) in the lower mevalonate pathway (Fig. 1). The excess addition of ATP is similar with previous study when acetyl-CoA was utilized as substrate for the in vitro production of isoprene [14].

### Quantitatively balancing enzyme levels to maximum the isoprene production

**Fig. 3:**
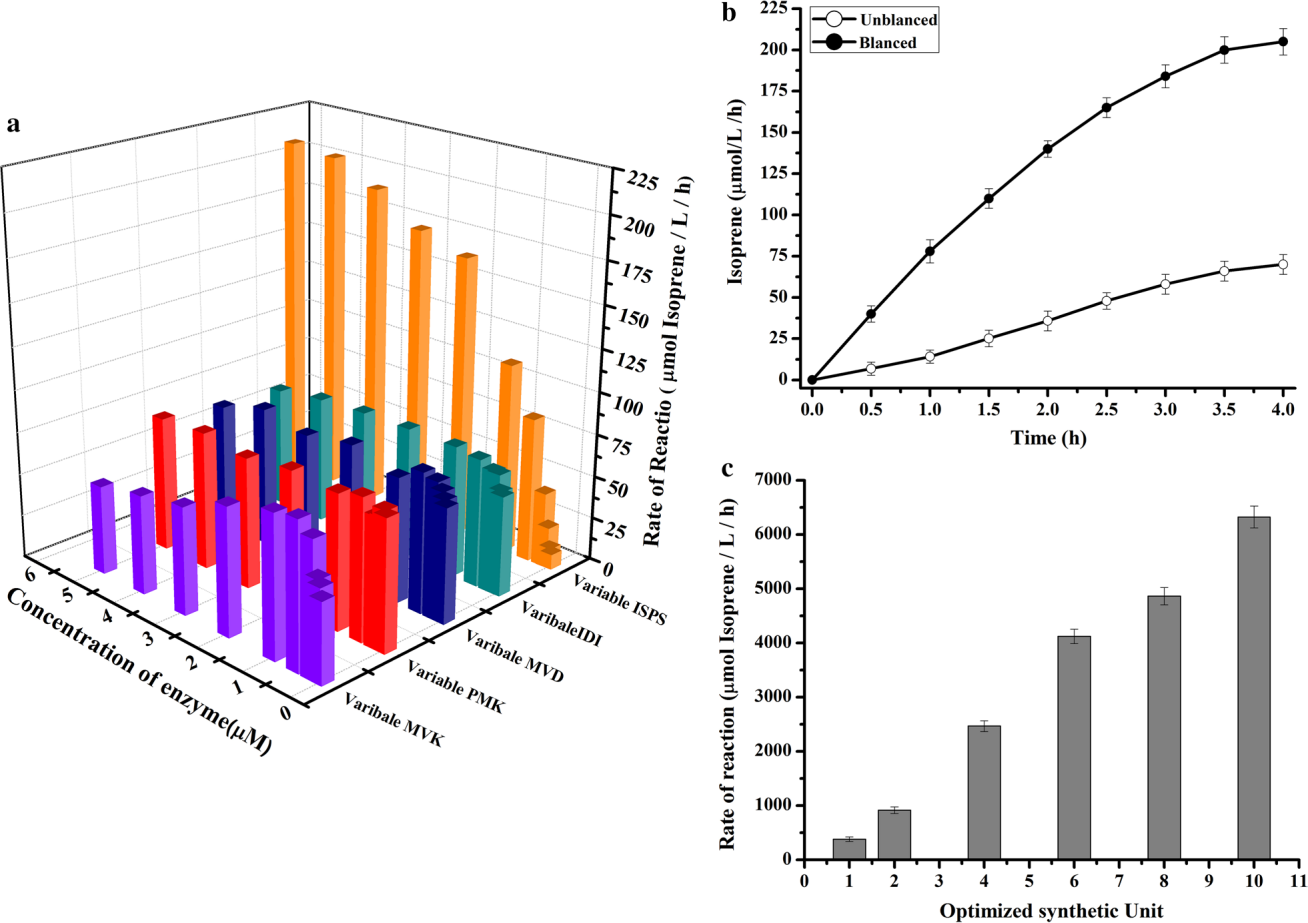
Optimization of the in vitro synthetic unit and the effects of enzyme levels on isoprene production. **a** Determination of the optimal enzyme level of each enzyme within synthetic unit, when four other enzymes were kept at constant level of 0.5 µM. **b** Production of isoprene using balanced or unbalanced synthetic unit. **c** Effects of increased synthetic units towards the isoprene production. Each unbalanced synthetic unit has 0.5 µM of MVK, PMK, MVD, IDI and ISPS. Each balanced synthetic unit has 0.5 µM of MVK, PMK, MVD; 1.0 µM of IDI, 8.0 µM of ISPS (MVK:PMK:MVD:IDI:Isps = 1:1:1:2:12). **a**, **b** and **c** followed the below conditions: 50 mM phosphate buffer, 30 mM potassium chloride, 10 mM magnesium chloride, and 4 mM β-mercaptoethanol, 10 mM ATP and 2.5 mM mevalonic acid. The data shown are means of three repeated experiments and the *error bars* present the standard deviation

In the in vitro isoprene biosynthesis system, the enzyme quantity can be precisely adjusted to optimize the flux through the biochemical steps comparing with the isoprenoids biosynthesis using living cells, in which promoter induction is often highly cooperative and fine control is difficult [11]. To quantitatively construct balanced in vitro system, we firstly screened the bottleneck enzymes which significantly affect the isoprene production. The effects of enzyme levels towards isoprene production were tested by varying the levels between 0.02 and 5 µM with the constant levels of other four enzymes at 0.5 µM (Fig. 3a) in the 2 ml in vitro system. The results showed that the enzymes had different effects towards the isoprene production. PMK and MVD were not belonging to the bottleneck enzymes in the pathway, as their levels did not significantly influence the isoprene production. For the enzymes of MVK and IDI, their increasing levels generated a 30–90% increase in isoprene production: MVK reached the maximum isoprene production at the level of 0.5 µM while IDI reached the maximum isoprene production at 1.0 µM (Fig. 3a). The last enzyme of ISPS, which catalyzes the production of isoprene from DMAPP, belonged to the bottleneck enzyme of this in vitro system, as its increasing levels from 0.02 to 5 µM gave a 30-fold increase in isoprene production. The results indicated that IDI and ISPS were two bottleneck enzymes in the in vitro production of isoprene from mevalonate, and their levels needed to be adjusted to balance the in vitro system. From the data of Fig. 1a, we could deduce that the optimized molarity ratio of MVK: PMK: MVD: IDI: ISPS was 1:1:1:2:16. One such balanced synthetic unit (0.5 µM of MVK, PMK, MVD; 1.0 µM of IDI, 8.0 µM of ISPS) could produce up to 382.4 µmol/L/h (26 mg/L/h) isoprene, which is nearly five folds of the isoprene production by the unbalanced synthetic unit (0.5 µM of each enzyme, Fig. 3b). Moreover, when multiple synthetic unit is supplemented in the 2 ml in vitro system, the production efficiency of isoprene could be significantly improved, 10 balanced synthetic units (5.0 µM of MVK, PMK, MVD; 10.0 µM of IDI, 80.0 µM of ISPS) would increase the isoprene production to 6323.5 µmol/L/h (430 mg/L/h) (Fig. 3c).

Our results demonstrated that balancing of enzyme levels could significantly increase the in vitro production of isoprene, which followed the results of in vivo studies that the balanced expression levels of heterologous enzymes is a key determinant in optimizing isoprenoid production [10, 11]. The in vivo studies aim to balance the heterologous pathways to reduce the growth inhibition effects towards the microbial hosts while the in vitro studies more focus on overcoming the limiting biochemical steps in heterologous flux towards the objective products. For example, we have noted that the enzymes levels of MVK did not apparently affect the in vitro production of isoprene (Fig. 3a) and they were kept at minimum level (0.5 µM) in our balanced synthetic unit. While in the in vivo studies for isoprene [3, 16] and isoprenoids biosynthesis [11], the expression of MVK were adjusted at higher levels, in which MVK was believed to be a key enzyme and was expressed by stronger promoters than PMK and MVD. We hypothesized that the in vivo system has specific mechanism to adjust the intra- and extracellular levels of mevalonate (MVA), and higher level of MVK helps to convert the intracellular MVA before it is transported off the cell. The last enzyme ISPS were both found bottleneck enzyme in previous study [17] as well as in this study. From the data of this study, ISPS catalyzed the rate-limiting biochemical step in heterologous flux from DMAPP to the isoprene. The in vivo models also supported this conclusion, it was proved that lower expression level of key enzyme downstream the IPP/DMAPP will lead to the accumulation of C5 building blocks (IPP and DMAPP), and will inhibit normal cell growth [9].

Comparing with the previous in vitro experiment [14] which incorporated as much as 12 enzymes of upper and lower MVA pathways, this study demonstrated a simpler system which only involved 5 enzymes and their levels were precisely adjusted to optimize flux through the biochemical steps. After optimization, the isoprene production was significantly improved (from 214.5 to 6323.5 µmol/L/h) comparing with the previous in vitro experiment [14].

### In vitro and in vivo production of isoprene from MVA: a comparison

**Fig. 4:**
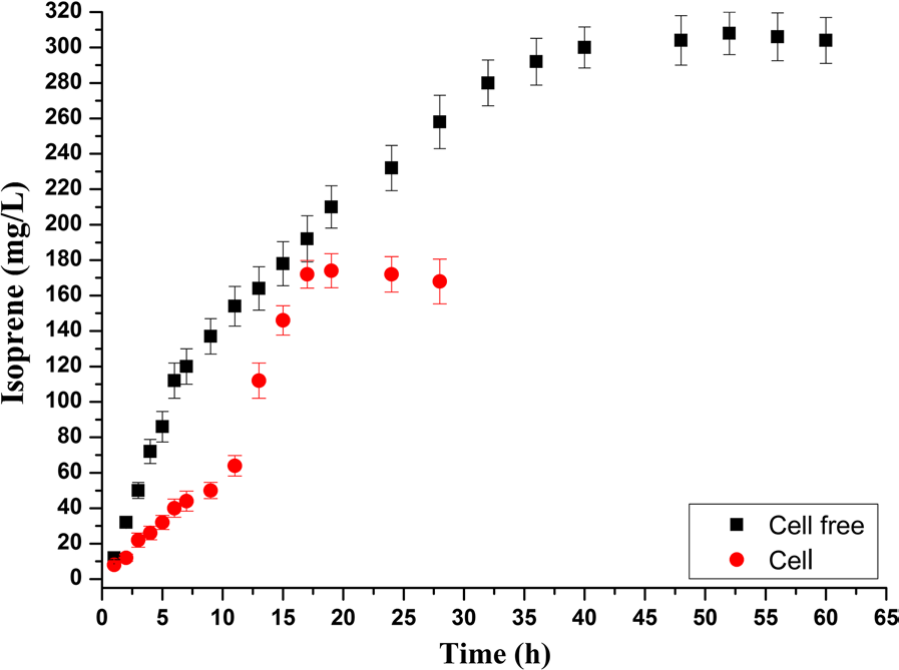
The comparison of isoprene production by 50 ml in vitro and in vivo systems. The concentration of MVK (0.5 µM) was similar in both in vitro (*black*) and in vivo (*red*) systems. The cell free in vitro system has 1 synthetic unit (0.5 µM of MVK, PMK, MVD; 1.0 µM of IDI, 8.0 µM of ISPS) with 50 mM phosphate buffer, 30 mM potassium chloride, 10 mM magnesium chloride, and 4 mM β-mercaptoethanol. 50 ml culture of engineered *E. coli* (BL21(DE3)/pYJM14/pACY-ISPS, Table 1) which expressed the five enzymes of the lower MVA pathway was utilized as the in vivo control. Both systems was initiated by addition of 2.5 mM mevalonic acid and incubated at 30 °C in rotary shaker (180 rpm). Samples were taken to analyze their isoprene production at different time points. The data shown are means of three repeated experiments and the *error bars* present the standard deviation

We further set up a 50 ml in vitro system and compared its isoprene production with the in vivo flask fermentation (50 ml), from the same starting concentration of mevalonate substrate. The results showed that the in vitro model apparently had higher isoprene production rate than the in vivo model during the earlier 12 h of isoprene production (Fig. 4). The initial isoprene production rate is about 220.6 µmol/L/h (15.0 mg/l/h, total 160 mg/l isoprene within 12 h), which is similar with the recent study for the in vitro isoprene production system [14]. Moreover, isoprene production remained longer for the in vitro system: from 20 to 40 h, the production of isoprene was suspended in the in vivo system, but was consistent in the in vitro system. Until 40 h, the production of isoprene from mevalonate by the in vitro system reached to 4442.4 µmol/L (302.0 mg/l). From the comparison of the isoprene production by the in vitro and the in vivo models, we could deduce that the balanced in vitro enzyme system led to higher production rate and prolonged isoprene production. We estimated that the unbalanced intracellular enzymes levels, accumulated toxic intermediates, competitive consumption of co-factors may affect the isoprene production by the in vivo methods.

## Conclusion

In this study, isoprene was produced from mevalonate by an optimized enzymatic process using the five recombinant enzymes of MVK, PMK, MVD, IDI and ISPS from the lower MVA pathway. Balancing and increasing the enzyme levels could apparently enhance isoprene production, indicated that the production rate could be further increased by raising the concentrations of the mevalonate and enzymes, which is hard to achieved by the in vivo approaches. When the balanced enzyme units were increased to ten, the proposed process would produce 6323.5 µmol/L/h (430 mg/L/h in a 2 ml system) isoprene from mevalonate. Moreover, this study showed that the proposed process has the advantages of longer producing period of 40 h comparing with the controlled in vivo process. The improved production efficiency indicated that the proposed strategy is useful for the enzymatic production of isoprene or isoprenoids.

## Methods

### Strains and plasmids

Bacterial strains and plasmids used in this study were listed in Table 1. *Escherichia coli* strain DH5α and BL21 (DE3) and *Saccharomyces cerevisiae* used in this study were purchased from Invitrogen. The *E. coli* DH5α strain and *E. coli* BL21 (DE3) were used for plasmids preparation and for protein overexpression respectively. *S. cerevisiae* was used for gene for cloning. *E. coli* DH5α and *E. coli* BL21 (DE3) were cultured in Luria–Bertani (LB) broth in construction of strains and plasmids. *S. cerevisiae* was cultured in YPD medium. Antibiotics were added at final concentration of 50 μg/mL for kanamycin, 34 μg/mL for chloramphenicol and 100 μg/mL for ampicillin when necessary.

### Protein preparation and purification

All PCRs were done using PrimerSTAR Max DNA polymerase (TAKARA, Dalian, China). Four genes of the enzymes MVK, PMK, MVD, IDI were amplified from *S. cerevisiae* genome (obtained from ATCC201508D) and cloned into the plasmid pET-30a(+). The plasmid pET-ERG12 was constructed by cloning the *ERG12* gene (for MVK) of *S. cerevisiae* into *Hind*III and *Xho*I sites of vector pET-30a(+) with primers ERG12_F and ERG12_R. The plasmid pET-ERG8 was constructed by cloning the *ERG8* gene (for PMK) from *S. cerevisiae* into *EcoR*I and *Xho*I sites of vector pET-30a(+) with primers ERG8_F and ERG8_R. The plasmid pET_ERG19 was constructed by cloning the *ERG*19 gene (for MVD) from *S.cerevisiae* into *Bam*HI and *Xho*I sites of vector pET-30a(+) with primers ERG19_F and ERG19_R. The fourth gene for enzyme IDI was amplified by PCR using primers IDI_F and IDI_R and cloned into *Bam*HI and *Xho*I sites of vector pET-30a(+). The resulting plasmid was named pET-IDI. The isoprene synthase (ISPS) from poplar was synthesized after code optimization and digested with enzymes *Bam*HI and *Xho*I, then ligated into the pET-30a(+), the resulting plasmid was named pET-ISPS (Table 1).

Individual recombinant enzymes were extracted and purified from the strains of *E.coli* BL21(DE3) harboring the relevant plasmids. The cultures were incubated in 200 ml LB medium with 50 µg/ml Kanamycin at 37 °C until the OD600 reached 0.6–0.8. The cultures were added 0.2 mM IPTG for induction and were cooled to 20 °C for protein expression. After further growth at 20 °C for 12 h, cells were harvested by centrifugation at 8000*g* and suspended in 10 mL 50 mM phosphate buffer (pH7.4) containing 4 mM β-mercaptoethanol. The suspension was lysed by sonication (at 60% output for 3 s pulses with 3 s intervals between each cycle) for 40 min at 4 °C with tube jacketed in wet ice and centrifuged at 18,000*g* for 10 min at 4 °C. The supernate filtered by 0.22 µm PALL filter was added into the Nickel Column, which was washed by 10 ml water and 10 ml binding buffer in order to ensure that the column was equilibrated, and then the protein containing 6 His-tag was able to specifically bind to nickel column. Unbound protein was washed out with 10 ml washing buffer 1, then washing Buffer 2 was used to wash any nonspecific binding protein. Elution buffer was added to wash the specific protein with collection of 10 ml fractions. The column was then re-equilibrated with buffer. Protein concentrations were measured with BCA protein assay kit using a spectrophotometer. Recombinant enzymes were stored at −80 °C after flash freezing in liquid nitrogen.

### In vitro reaction system

The reaction system was performed as previously described [18] to ensure the correct concentration of individually enzyme. For the demonstrated assay, a variety levels of each enzyme component and ATP were added to the reaction buffer which contain 50 mM potassium phosphate, 30 mM potassium chloride, 10 mM magnesium chloride, and 4 mM β-mercaptoethanol. The reaction was initiated by addition of 2.5 mM mevalonic acid which was made by the saponification of mevalonolactone with KOH at 1.05:1 (vol:vol) KOH:mevlonolactone for 30 min at 37 °C, and then incubated at 30 °C for 4 h. To confirm isoprene had accumulated in the 2 ml reaction system, 0.2 ml of the headspace gas of sealed 10 ml vial were analyzed by gas chromatography using Agilent 7890B GC (Agilent, American) equipped with a flame ionization detector and a Agilent HP-INNWOX column, designed to detect short-chain hydrocarbons. Amounts of isoprene produced in the recombinant system were calculated by comparison with an isoprene standard (Aladdin, China).

### Gas chromatography (GC) analysis of isoprene

1 ml of off-gas samples from the headspace of the fermentor were analyzed as described earlier [16] using a GC (Agilent 7890A, America) equipped with a flame ionization detector (FID) and a HP-INNOWAX column (30 m × 320 μm × 8 μm). N_2_ was used as carrier gas with a linear velocity of 1 ml/min. The product was characterized by direct comparison with standard isoprene (TCI-EP, Tokyo, Japan). The peak area was converted to isoprene concentration by comparing with a standard curve plotted with a set of known concentration of isoprene. Then isoprene accumulation was measured every 30 min by GC.

### The comparison of production of isoprene in vitro and in vivo

To compare isoprene production in shake flasks (in vivo) and in vitro, the engineered strain *E. coli* BL21(DE3)/pYJM14/pACY-ISPS (Table 1) were cultured in 500 ml sealed glass flasks containing 50 ml of M9 medium supplemented with 10 g/L glucose and 0.5 g/L yeast extract, 34 μg/mL chloramphenicol, and 100 μg/mL Ampicillin. IPTG was added to the medium when the cell density OD_600_ reached to 0.6 and the cultures were performed at 30 °C in rotary shaker (180 rpm). For isoprene production, the substrate 2.5 mM mevalonate was added after the cell was induced and cultivated at 30 °C for 4 and 16 h respectively. During the cultivation, samples of both the flask headspace and the culture were taken at multiple times points.

To set up similar enzyme levels in the in vitro system comparing to the in vivo control. The intracellular MVK concentration of the in vivo strain was firstly quantified using Quantity One Software (Biorad) described previously [19]. When OD_600_ reached 0.6, intracellular MVK concentration was about 0.4 µM. According to the optimized molarity ration for the in vitro system, 0.4 µM of MVK, PMK, MVD, 0.8 µM of IDI, 6.4 µM of ISPS was added in the 50 ml in vitro reaction system. 10 mM ATP, 50 mM potassium phosphate, 30 mM potassium chloride, 10 mM magnesium chloride, and 4 mM β-mercaptoethanol were also added in the reaction system. The reaction was initiated by addition of 2.5 mM mevalonic acid which was made by the saponification of mevalonolactone with KOH at 1.05:1 (vol:vol) KOH:mevlonolactone for 30 min at 37 °C, and then incubated at 30 °C in rotary shaker (180 rpm). During the cultivation, samples of both the flask headspace and the culture were taken at multiple times points.

## Abbreviations

MVA pathway: mevalonate pathway
MEP pathway: methylerythritol 4-phosphate pathway
MVK: mevalonate kinase
PMK: phosphomevalonate kinase
MVD: diphosphomevalonaet decarboxylase
IDI: isopentenyl diphosphate isomerase
ISPS: isoprene synthase
IPTG: isopropyl-β-d-thiogalactoside
GC: gas chromatography
